# Case Report: Diagnosis and treatment strategy for a case of multiple chest wall implantations and neck lymph node metastasis following total areola endoscopic surgery for papillary thyroid carcinoma

**DOI:** 10.3389/fonc.2026.1692528

**Published:** 2026-02-24

**Authors:** Shuai Zhang, Qizhi Li, Liang Jiang, Xiqun Zhu, Xiaohui Jiang

**Affiliations:** 1 Department of Head and Neck Surgery, Hubei Cancer Hospital, Tongji Medical College, Huazhong University of Science and Technology, Wuhan, Hubei, China; 2 Department of Gynecologic Oncology, Hubei Cancer Hospital, Tongji Medical College, Huazhong University of Science and Technology, Wuhan, Hubei, China

**Keywords:** anlotinib, case report, chest wall implantations, endoscopic thyroid surgery, neck lymph node metastasis, tall cell variant of papillary carcinoma

## Abstract

**Background:**

Endoscopic thyroid surgery has gained increasing prominence in the field of thyroid surgery. However, special attention should be paid to its limitations in total thyroidectomy, surgical tract implantation metastasis, and suboptimal cervical lymph node dissection. This case report describes the occurrence of chest wall implantations and neck lymph node metastases in an elderly patient following total areola endoscopic surgery for papillary thyroid carcinoma (PTC), emphasizing the need for appropriate diagnostic and therapeutic interventions in such cases.

**Methods:**

The patient underwent comprehensive preoperative evaluation, including neck ultrasound and chest computed tomography (CT), followed by neck lymph node dissection and resection of the chest wall implantation lesions. The patient was then initiated on oral anlotinib therapy and scheduled for regular clinical follow-up, without undergoing genetic testing.

**Results:**

We present a case involving a 65-year-old female who underwent total areola endoscopic surgery for PTC. Due to postoperative laryngeal stridor and intermittent respiratory distress, radioactive iodine-131 therapy was not pursued, respecting the preferences of the patient and her family. During this period, multiple chest wall implantations were identified and excised under local anesthesia. Pathological examination revealed a transition from classic PTC to the tall-cell variant. Despite further neck lymph node metastasis and recurrent chest wall implantation, the family declined general anesthesia surgery. Anlotinib was administered. Follow-up showed a reduction in the size of neck and chest wall lesions, with significant pain relief.

**Conclusion:**

Careful preoperative assessment is essential to appropriately select patients for Total areola endoscopic thyroid surgery. In elderly patients with postoperative local lymph node recurrence, surgical tract metastasis, vocal cord paralysis, mild dyspnea, patient and family refusal of further treatment, or progression to the more aggressive tall cell variant of PTC without prior radioiodine therapy, oral administration of anlotinib may be considered after thorough discussion. This targeted therapy may result in tumor regression, symptom amelioration, and potentially extended overall survival in such challenging cases.

## Introduction

The incidence of thyroid cancer has risen globally in recent years. Differentiated thyroid carcinoma, the most prevalent subtype, exhibits a favorable prognosis and long-term survival rate (1, 2). Surgery remains the primary treatment, yet traditional open thyroidectomy often leaves visible neck incisions or scars, potentially impairing patient self-perception and quality of life, particularly among young and female individuals (3, 4). In response, the advancement of medical technology has facilitated the widespread clinical adoption of endoscopic thyroid surgery, which offers the advantages of concealed incisions and superior cosmetic outcomes (5).

Endoscopic thyroid surgery has progressively matured and become standardized, giving rise to various technical approaches, including the endoscopic oral approach (EOA), endoscopic gasless axillary approach (EGAA), endoscopic bilateral areola approach (EBAA), minimally invasive video-assisted approach (MIVAA), and endoscopic bilateral axillo-breast approach (EBABA). Each technique has its own advantages and limitations (5–7). Recent studies indicate that the total areolar approach, due to its relatively lower technical complexity, is more accessible for surgeons, thus becoming the most prevalent technique (5). However, concerns persist about the safety and reliability of these endoscopic methods in thyroid cancer surgery, particularly regarding severe complications such as the adequacy of central lymph node dissection, local recurrence, and tumor implantation (5, 8).

We report a case of endoscopic thyroidectomy via the total areola approach for thyroid nodules, with postoperative pathology confirmed PTC. During follow-up, chest wall metastases were excised under local anesthesia. Recurrent neck lymph node metastasis and chest wall implantation prompted a recommendation for surgery under general anesthesia, which the patient’s family declined. Anlotinib, a novel TKI, has been reported to demonstrate significant efficacy in a patient with radioiodine-refractory differentiated thyroid cancer (RAIR-DTC) (9). To administer anlotinib can reduce the the neck and chest wall lesions, along with significant pain relief. This case highlights a rare instance of managing chest wall implantations and neck lymph node metastasises without iodine-131 therapy, where anlotinib was effective despite the absence of genetic testing, resulting in tumor regression and ongoing symptomatic improvement.

## Case report

In April 2018, a 67-year-old female patient with thyroid nodules underwent an endoscopic right thyroidectomy and isthmusectomy via the total areola approach, along with a subtotal left thyroidectomy at an external hospital. Postoperative routine pathological examination of the right thyroid gland and isthmus revealed classic-type papillary thyroid carcinoma (PTC) with a single focus. The tumor, measuring 2.2 cm in maximum diameter, was located adjacent to the thyroid isthmus. No evidence of carcinoma metastasis was found in the right central compartment lymph nodes (0/3). Although the tumor invaded the thyroid capsule, it did not extend into the strap muscles, and there was no indication of vascular, lymphatic, or perineural invasion. Pathological examination of the left subtotally resected thyroid gland indicated the presence of nodular goiter. The postoperative pathological stage was classified as T2N0M0. She experienced hoarseness, choking while drinking, and numbness in her hands. Over time, her hoarseness has gradually worsened. she has been consistently on thyroid hormone replacement therapy and regular follow-up examinations.

In March 2019, a follow-up neck ultrasound at an external hospital identified multiple enlarged lymph nodes in the right neck regions IV and VI, indicating potential lymph node metastasis. A subsequent fine-needle aspiration cytology at another hospital confirmed papillary thyroid cancer infiltration.

**Figure 1:**
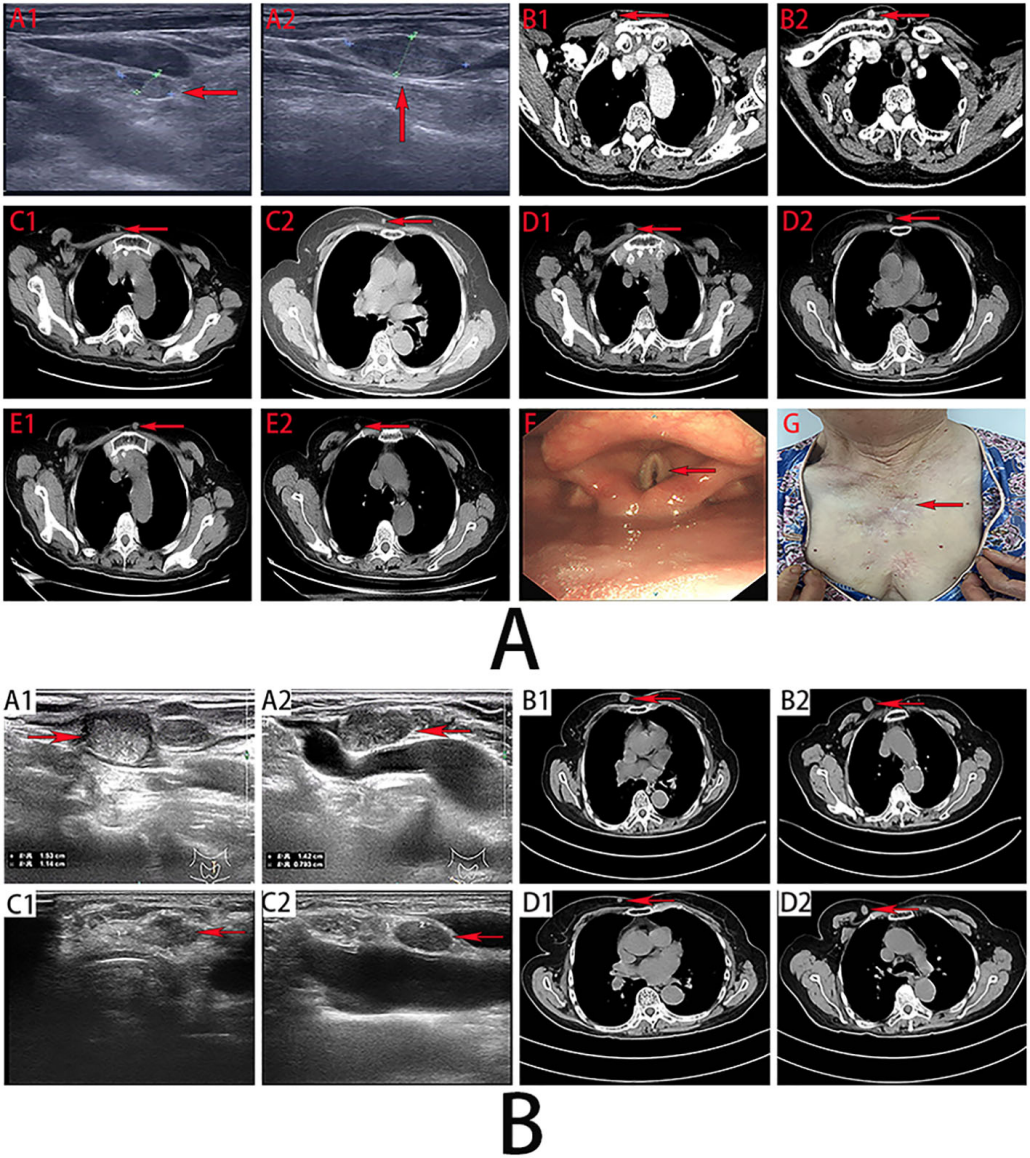
**(A)**. Preoperative imaging diagnosis. (A1, A2) (March 2019): Lymph node metastases of PTC in the right neck (level III) and left neck (level IV). **(B1, B2, C1, C2, D1, D2, E1, E2)**, Chest wall implantations of PTC in November 2020, March 2021, and October 2021, respectively. **(F)** Laryngoscopy (July 2024) shows poor abduction of bilateral vocal cords. **(G)** Postoperative presentation of multiple implantation and metastatic lesions on the chest wall and neck. **(B)**. Ultrasonographic images of metastatic lymph nodes in the neck of PTC before and after oral administration of anlotinib (**A1, A2** vs **C1, C2**); CT images of chest wall implantation lesions before and after oral administration of anlotinib (**B1, B2** vs **D1, D2**).

In April 2019, specialist examination revealed a firm, enlarged lymph node measuring approximately 1.5×1 cm in the right cervical level III. neck ultrasound at our hospital identified the left level IV lymph node with indistinct corticomedullary differentiation, measuring approximately 0.86×0.47cm, and an enlarged right level III lymph node measuring approximately 1.87×0.82cm (**Figures 1A–A2**). The patient subsequently underwent left thyroid remnant resection and bilateral neck lymph nodes dissection. Postoperative pathology revealed lymph nodes metastasis of PTC in 9 of 34 lymph nodes: 3/9 in left levels III、IV, 1/15 in right level II, 4/6 in right level III, and 1/4 in right levels IV、V. Postoperatively, the patient was classified as high risk for recurrence and was advised to undergo radioactive iodine-131 therapy. The patient and family were clearly informed that the iodine-131 ward is an isolation unit and that the patient must be fully self-sufficient. They were also told that administration of iodine-131 can cause symptoms such as nausea and vomiting, which may worsen dyspnea and, in rare cases, necessitate emergency tracheotomy. The patient and family nevertheless declined the treatment.

**Figure 2:**
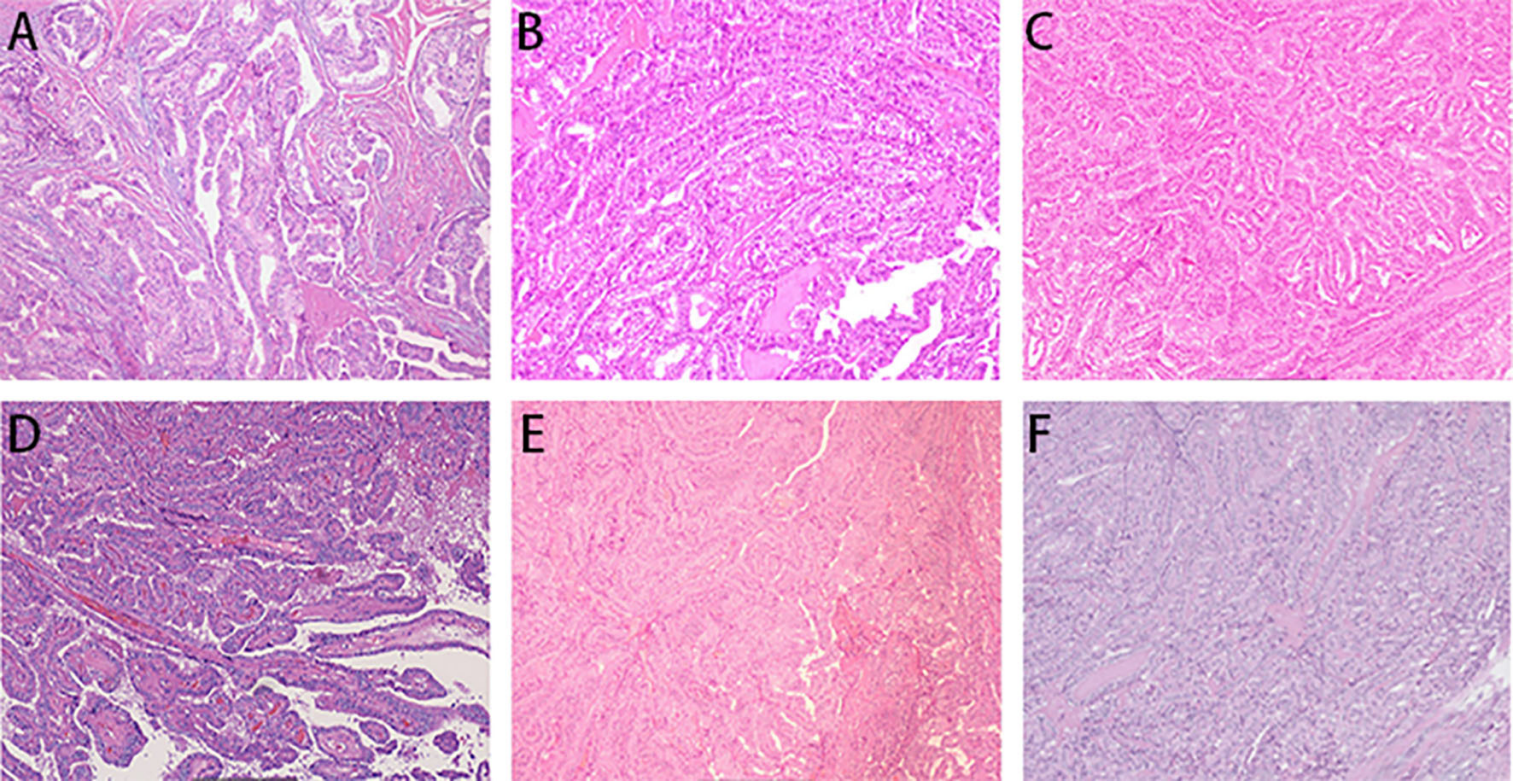
Histopathological examination. Pathological examination after multiple surgeries for chest wall implantations of PTC **(A–F)**.

In September 2019, the patient incidentally discovered a lesion on the anterior chest wall. On December 17, 2019, the patient returned to our hospital. During the physical examination, multiple palpable masses were detected on the chest wall, the largest measuring about 0.8×0.3 cm. We conducted a fine-needle aspiration cytology examination on the chest mass, which confirmed the diagnosis of metastatic papillary thyroid carcinoma. In December 2019, a fine-needle aspiration biopsy of the chest lesion revealed cytomorphological features consistent with metastatic PTC. Subsequently, the patient underwent local anesthetic excision of the chest wall lesion. Postoperative pathological examination confirmed the presence of papillary thyroid carcinoma within fibrotic connective and muscle tissue (**Figure 2A**).

In February 2020, a right chest wall lesion was detected again, which showed slow progression. Chest CT in November 2020 revealed chest wall lesions measuring approximately 0.72×0.61cm and 1.05×0.84cm (**Figures 1A, B1, B2**). Subsequently, the patient underwent chest wall lesions resection. Postoperative pathological examination demonstrated: PTC infiltration observed in muscle and fibrous tissues (**Figure 2B**). Immunohistochemistry: BRAF (V600E) (+).

In March 2021, a right chest wall lesion was detected again, exhibiting slow growth and mild pain. Chest CT scans revealed lesions measuring 0.63×0.49 cm and 0.52×0.42 cm (**Figure 1A, C1, C2**). By October 2021, the lesions had enlarged with significant pain. Follow-up CT showed the lesions had grown to 1.25×0.89 cm and 0.93×0.75 cm (**Figures 1A, D1, D2**). The patient then underwent resection of the chest wall lesions. Postoperative pathology identified PTC within fibrotic connective and muscle tissue (**Figure 2C**). Immunohistochemistry showed BRAF (V600E) (+), TTF-1 (+), and TG (+).

In February 2022, a lesion on the right chest wall accompanied by mild pain was detected. By April 2022, the patient underwent resection of the chest wall lesion. Postoperative pathological analysis identified PTC infiltration within fibrous connective tissue (**Figure 2D**). Immunohistochemistry results showed TG (+) and BRAF (V600E) (+).

In December 2022, multiple chest wall lesions reappeared, accompanied by mild pain. By April 2023, chest CT scans identified lesions measuring approximately 1.11×0.58 cm and 0.73×0.62 cm (**Figures 1A, E1, E2**). Chest wall lesions resection were subsequently. Postoperative pathological analysis revealed PTC infiltration within fibrous connective tissue(**Figure 2E**). Immunohistochemistry showed TG (+) and TTF-1 (+).

In August 2023, chest wall lesions were identified, accompanied by mild pain and progressive enlargement. Surgical excision under local anesthesia occurred in October 2023. Postoperative pathology indicated PTC infiltration in the subcutaneous fibrous connective tissue. Microscopic examination diagnosed the lesions as tall cell variant of papillary carcinoma with mitotic figures approximately 3–5 per 2 mm² and no tumor necrosis (**Figure 2F**).

**Figure 3:**
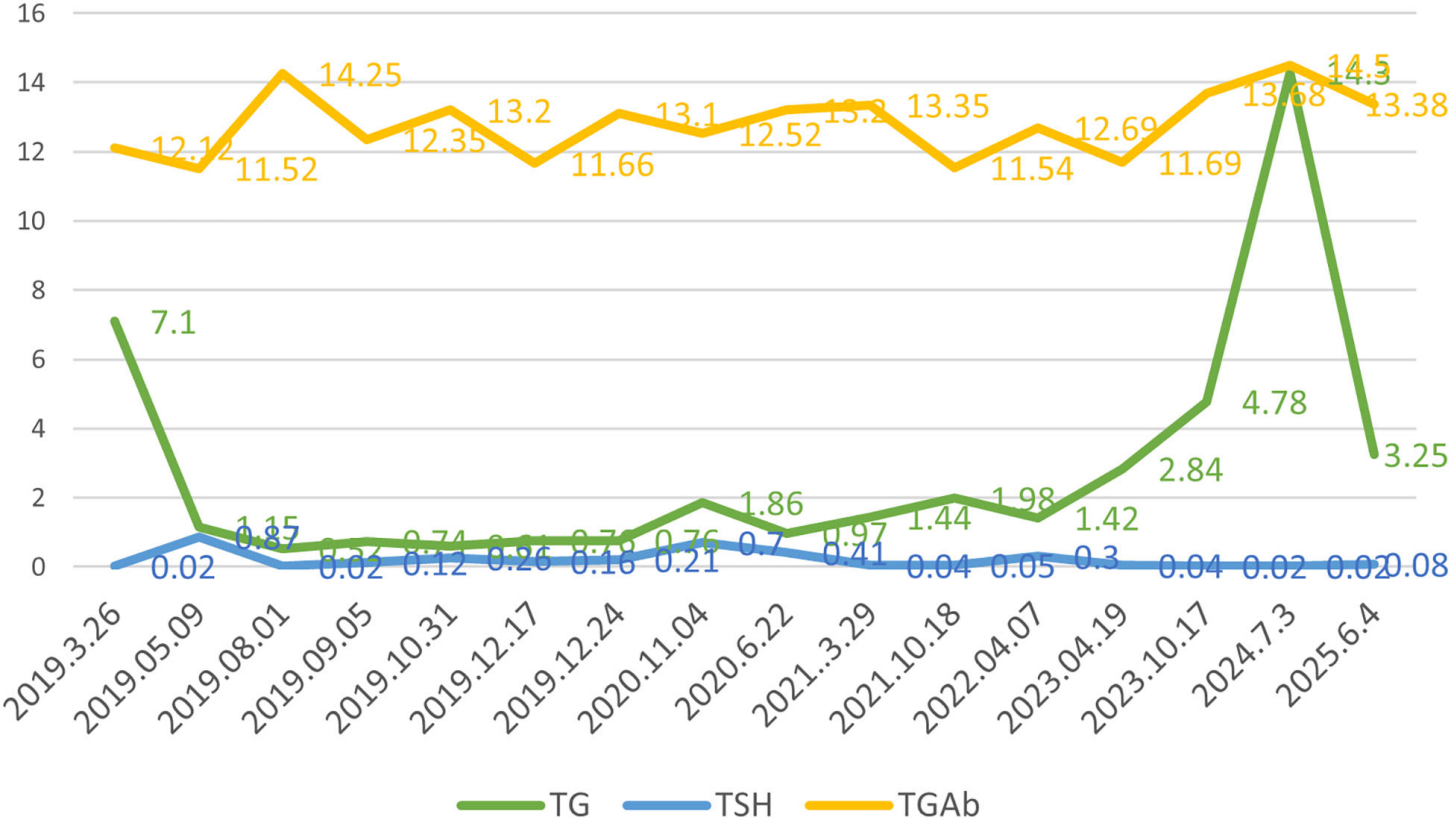
Laboratory test indicators of thyroglobulin (TG) (ng/ml), thyroid-stimulating hormone (TSH) (mIU/L), and anti-thyroglobulin antibody (TGAb) (IU/ML) in the patient during the follow-up period.

In March 2024, recurrent lesions were identified in the anterior neck region and chest wall, enlarging progressively with marked tenderness. By July 2024, the patient exhibited persistent hoarseness and intermittent dyspnea (**Figures 1A, F**). Neck ultrasonography and chest CT confirmed recurrent neck lymph node metastasis and chest wall implantation. Abnormal lymph nodes measured approximately 1.53×1.14 cm in the left level VI and 1.42×0.79 cm in the right level IV (**Figures 1B, A1, A2**). Two chest wall lesions measured approximately 1.67×1.10 cm and 2.41×1.51 cm (**Figure 1B, B1, B2**). Surgery under general anesthesia was advised, but the family declined. With informed consent, oral anlotinib was administered. Follow-up showed regression of neck and chest wall lesions (**Figures 1A, G**), with significant pain relief. By June 2025, neck ultrasonography and chest CT indicated the left level VI and right level IV lymph nodes had reduced to about 1.24×0.78cm and 1.01×0.56cm, respectively (**Figures 1B, C1, C2**). The chest wall lesions measured approximately 0.74×0.45cm and 1.26×.89cm (**Figures 1B, D1, D2**). The patient remains clinically stable on regular anlotinib therapy, with partial thyroid function parameters under ongoing monitoring (**Figure 3**).

## Discussion

Endoscopic thyroidectomy offers the primary advantage of relocating the surgical incision to a less visible area, thereby significantly improving cosmetic outcomes. The trans-areolar endoscopic approach is prevalent due to its relative ease of execution (5). However, this procedure is primarily cosmetic rather than minimally invasive, appealing mainly to patients with cosmetic concerns, especially younger and female patients (4). Due to the constrained operative space and the high technical demands, strict adherence to surgical indications is crucial (5). The case presented highlights the potential limitations of endoscopic thyroid surgery, particularly in elderly patients. The patient described in this report is an elderly female. Notably, the initial treating hospital performed endoscopic thyroid surgery without obtaining fine-needle aspiration cytology. Even if the patient and her family consented to endoscopic surgery, they should have been explicitly informed that the nodule could be malignant, that this uncertainty might lead to an inappropriate choice of surgical approach, and that a reoperation could be required shortly after the procedure. Moreover, endoscopic approaches create an extended subcutaneous tract, which could facilitate implantation metastasis during thyroid cancer surgery. Consequently, the appropriateness of endoscopic thyroidectomy for this patient is questionable. This case also provides a clear warning to surgeons: we must strictly observe indications for endoscopic thyroid surgery and comprehensively disclose surgical risks and potential complications to patients. Only by doing so can we fulfill our responsibility to patients and show proper respect for the disease. Lymph node metastasis is a well-recognized risk factor for recurrence in PTC (10). However, the efficacy of endoscopic techniques for comprehensive central lymph node (CLN) dissection remains contentious. Previous studies have reported that a substantial proportion of surgeons using transareolar (66.78%) and transaxillary (40.46%) approaches consider CLN dissection to be incomplete. This may be attributed not only to surgeon experience but also to the presence of metastatic lymph nodes behind the sternum, which can create visual blind spots during the breast-based approaches. Consequently, rigorous preoperative evaluation of neck lymph node status is essential for appropriate patient selection and surgical planning (5, 11).Fine-needle aspiration biopsy (FNAB) remains the gold standard for diagnosing benign and malignant thyroid nodules and lymph node metastasis (2).Nevertheless, false negatives occur, necessitating thyroid-related genetic testing for nodules with high malignancy suspicion. Ultrasonography offers high specificity but variable sensitivity, particularly struggling to detect deep neck and mediastinal lymph node metastases (2). Studies suggest the contrast-enhanced neck CT provide superior predictive capability for neck lymph node metastasis (12). The 2025 ATA Guidelines for Adult Differentiated Thyroid Cancer recommend ultrasound as the primary imaging method for assessing lymph nodes in the thyroid, central neck, and lateral neck regions. For patients suspected of having advanced or aggressive differentiated thyroid cancer, it is strongly advised to complement physical examination and ultrasound with enhanced neck CT and MRI. Enhanced CT, in particular, offers higher sensitivity than ultrasound for evaluating central and lateral neck lymph nodes, thereby reducing the likelihood of missed lymph node metastases and aiding in surgical planning. MRI, while not exposing patients to ionizing radiation, also has a contrast agent with lower nephrotoxicity compared to CT’s. Despite these differences, CT and MRI with contrast agents are noted to have similar effectiveness in detecting neck lymph node metastasis (13). We identify cases where the tumor is confined to the thyroid and imaging shows no lymph node metastasis as key candidates for endoscopic thyroid surgery (5). One year post-total areola endoscopic thyroid surgery, the patient developed central and lateral neck lymph node metastases, suggesting inadequate preoperative lymph node evaluation. For novices in endoscopic techniques, unlike open thyroid surgery, the aerosol from ultrasonic scalpels and improper energy device handling could heighten the risk of recurrent laryngeal nerve (RLN) and parathyroid gland injuries. Intraoperative neuromonitoring and negative parathyroid imaging can mitigate these risks (14, 15). No significant difference in RLN injury types exists between open thyroidectomy and endoscopic approaches, with injuries mainly due to traction, clamping, compression, contusion, pressure, and thermal damage (16). In this case, RLN injury may be attributed to the surgeon’s inexperience in endoscopic thyroid surgery, lack of intraoperative neuromonitoring, and anatomical variations of the right RLN.

Differentiated thyroid cancer patients at heightened risk of recurrence commonly undergo surgical resection followed by radioactive iodine (RAI) therapy to eliminate residual tumor and prevent disease relapse, particularly for advanced or metastatic cases (17). However, in the present case, an elderly individual exhibiting intermittent respiratory distress and pronounced nocturnal laryngeal stridor, did not receive additional iodine-131 treatment following comprehensive evaluation and discussion with the patient and family. Instead, the patient was prescribed oral thyroid hormone for thyroid-stimulating hormone (TSH) suppression therapy. Post-thyroidectomy, TSH suppression therapy is essential in managing thyroid cancer to prevent tumor progression or recurrence. The American Thyroid Association (ATA) guidelines recommend a TSH target of 0.1–0.5 mIU/L, adjusted for tumor recurrence risk and patient age (18). Cancer invasion disrupts thyroid architecture, causing follicular epithelial cells to release excess thyroglobulin(TG) into the bloodstream, raising serum TG levels. However, thyroglobulin antibodies can affect TG levels (19). Since May 9, 2019, the patient has been monitored for TG and TSH, with thyroglobulin antibodies consistently normal, minimally affecting TG levels.

Endoscopic thyroid surgery, compared to traditional open surgery, raises concerns about tumor implantation along the surgical pathway, where thyroid tissue or lesions might implant following remote-access thyroidectomy. Factors contributing to this risk include direct contamination of the surgical field, shedding and aerosolization of tumor cells, excessive tumor manipulation, tumor biological characteristics, and the surgeon’s technical skills (20, 21). Research indicates that tumor size may significantly affect tract recurrence rates. Consequently, during endoscopic procedures, surgeons must carefully handle the thyroid to maintain capsule integrity and ensure complete specimen resection (21).

Postoperative multiple chest wall implantation in this patient may be linked to intraoperative tumor capsule rupture, surgical route contamination by tumor cells, and inadequate surgical skill. Despite multiple resections of chest wall implantation lesions with negative surgical margins, recurrent lesions emerged at various chest wall sites approximately a year apart. The mechanisms driving this phenomenon remain unclear. Literature suggests preventive measures to reduce postoperative implantation metastases: careful handling and isolation of nodules, avoiding surface tears, using retrieval bags for excised specimens, and irrigating the surgical field to minimize exfoliated cells. An appropriate cytocidal solution should be used for irrigation (21).

We present a rare case of pathological transition from classical PTC to the tall cell variant (TCV) following multiple resections of chest wall implantation lesions. TCV constitutes 2%–19% of PTC cases and is characterized by larger tumors, extrathyroidal extension, and a prevalence in patients aged 50–60y. It frequently metastasizes to lymph nodes and distant sites, with BRAF mutations in 80%–100% and TERT promoter mutations in about 30% of cases (22). Radioactive iodine-131 therapy does not appear to enhance cancer-specific survival for TCV-PTC (23). The patient opted against radioiodine therapy for personal reasons. Beyond standard treatments, novel targeted therapies, such as tyrosine kinase inhibitors (TKIs), have shown efficacy in managing specific thyroid cancer types, particularly with distant metastasis and disease progression. Anlotinib, a novel TKI inhibitor, has shown significant efficacy in a patient with RAIR-DTC possessing concurrent TERT promoter and BRAF mutations (9, 17). Following comprehensive communication, the patient commenced regular oral anlotinib treatment. During follow-up, there was notable regression of neck lymph node metastases and chest wall implantations, along with pain relief, without any uncontrollable drug-related adverse effects. The patient experienced substantial clinical benefits.

**Figure 4:**
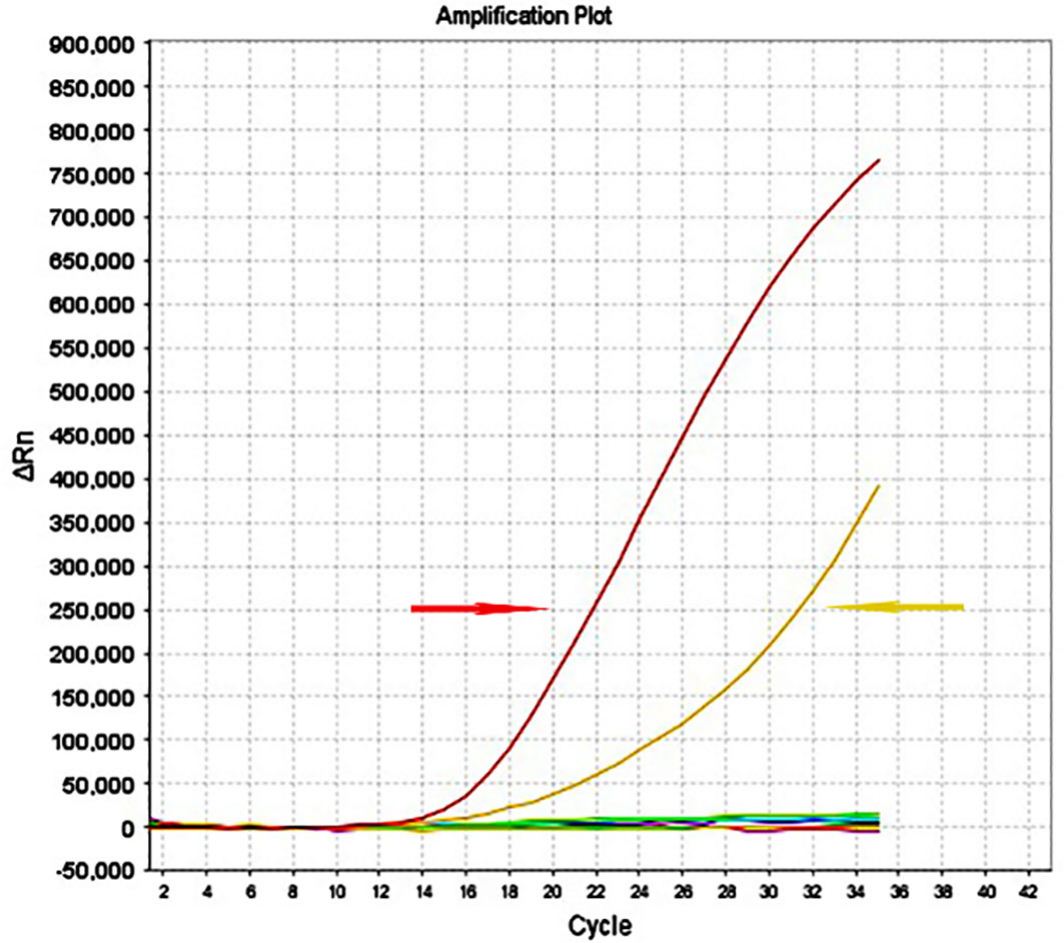
Gene mutations of BRAFV600E (Red arrow) and the TERT promoter (Yellow arrow) in cervical metastatic lymph nodes and chest wall implantation lesions.

This report presents several limitations. Firstly, the surgery and fine-needle aspiration cytology were conducted at different hospitals, leading to partial data loss. Secondly, two excisions of the chest wall lesions were performed at our hospital without prior chest CT or ultrasonography. Thirdly, no genetic testing related to thyroid cancer was performed before administering anlotinib. Fourthly, given the high likelihood of thyroid cancer recurrence and metastasis in this patient, and considering the patient’s and family’s refusal of iodine-131 treatment due to potential tracheotomy, it is crucial to offer a thorough professional evaluation of vocal cord movement and respiration. Fifthly, because the patient is of advanced age, the contrast agent used in enhanced CT scans could significantly affect renal function; therefore, MRI is likely a more suitable alternative. Sixthly, it is important to acknowledge that combining radioactive iodine whole-body imaging with enhanced CT or MRI offers a more comprehensive assessment of potential metastasis in cervical lymph nodes and other areas. Seventhly, with the informed consent of the patient and their family, an 8-gene panel test for thyroid cancer was performed on the resected specimen of a chest wall implant metastasis obtained in October 2023, which was pathologically diagnosed as high-grade differentiated thyroid carcinoma (papillary carcinoma, tall cell variant) with a mitotic count of approximately 3–5 mitoses per 2 mm² and no evidence of tumor necrosis. Mutations were identified in the *BRAF* gene and the TERT promoter (**Figure 4**).

## Conclusion

Endoscopic thyroidectomy using the complete areolar approach is well-established in clinical practice but demands skilled surgeons for optimal results. Post-surgery, instances of neck lymph node and surgical tract implantation have been noted. Subsequent surgeries and pathological assessments revealed a shift from classical PTC to the tall cell variant. Considering the patient’s advanced age and risk of severe complications like dyspnea, oral anlotinib was prescribed following comprehensive discussions and informed consent with the patient and family. This treatment has the potential to significantly reduce tumor size and alleviate clinical symptoms.

## Data Availability

The original contributions presented in the study are included in the article/supplementary material. Further inquiries can be directed to the corresponding author.
